# Remimazolam Anaphylaxis during Induction of General Anesthesia Confirmed by Provocation Test—A Case Report and Literature Review

**DOI:** 10.3390/medicina59111915

**Published:** 2023-10-30

**Authors:** Sangho Lee, Joyoung Park, Na Hei Kim, Halin Hong, Kyoung Hee Sohn, Hee Yong Kang, Mi Kyeong Kim, Ann Hee You

**Affiliations:** 1 Department of Anesthesiology and Pain Medicine, Kyung Hee University College of Medicine, Kyung Hee University Hospital, Seoul 02447, Republic of Korea; sangholee@khu.ac.kr (S.L.); joding107@gmail.com (J.P.); naheikim@gmail.com (N.H.K.); hongss21@gmail.com (H.H.); ujuabba@gmail.com (H.Y.K.); mkanes@gmail.com (M.K.K.); 2 Division of Pulmonary and Allergy, Department of Internal Medicine, Kyung Hee University Hospital, Seoul 02447, Republic of Korea

**Keywords:** dextran 40, general anesthesia induction, intradermal test, literature review, midazolam, remimazolam, perioperative anaphylaxis, provocation test, tryptase

## Abstract

*Background*: Remimazolam besylate, a newly developed drug, is linked to various anaphylaxis cases. We present a case of remimazolam anaphylaxis confirmed using a provocation test. *Case*: A 51-year-old female patient was scheduled for humeral pinning. General anesthesia was induced using remimazolam, rocuronium, and remifentanil. After tracheal intubation, the patient experienced decreased blood pressure, increased heart rate, and a systemic rash. Epinephrine was administered repeatedly, and the patient’s vital signs stabilized. Acute phase tryptase levels were within normal limits. After four weeks, intradermal test results were negative. When remimazolam was administered intravenously for the provocation test, facial swelling, flushing, and coughing occurred, which improved with epinephrine. The culprit drug was identified as remimazolam using a provocation test. *Conclusions*: When anaphylaxis occurs during anesthesia induction, remimazolam should not be ruled out as the causative drug. If the skin test result for remimazolam is negative, a provocation test should be considered. The provocation test should be initiated cautiously at a low dose under careful patient monitoring.

## 1. Introduction

Perioperative anaphylaxis occurs in approximately 1 in 10,000–20,000 cases of general anesthesia, with a 3–9% mortality rate [1]. Anaphylaxis is a severe, acute type 1 hypersensitivity reaction that requires immediate epinephrine, real-time blood pressure monitoring, airway security, and fluid administration [2]. Remimazolam besylate is a relatively recently developed ultra-short-acting benzodiazepine used for the induction and maintenance of sedation and general anesthesia [3]. Its use is gradually increasing owing to its rapid onset, offset, and hemodynamic stability [4]. Similar to midazolam, the effects of remimazolam can be reversed using flumazenil, and it offers a rapid recovery from general anesthesia [5]. It causes less respiratory depression compared to traditional sedative drugs such as propofol [6,7]. Additionally, while remimazolam is classified as a benzodiazepine, previous studies have reported that it does not increase the risk of postoperative delirium [8,9]. Although the safety of remimazolam has been evaluated, various cases of remimazolam anaphylaxis have been recently reported [10,11,12,13,14,15].

In this case report, anaphylaxis occurred during the induction of general anesthesia. Subsequent allergy tests showed negative results for all intradermal tests. However, a provocation test confirmed remimazolam as the causative drug with a positive result.

## 2. Case Report

Written informed consent was obtained from the patient for this case report. Ethical approval was obtained from the Institutional Review Board of Kyung Hee University Hospital (KHUH 2023-10-003) on 5 October 2023. A 51-year-old female patient (height 170 cm; weight 52 kg) was scheduled to undergo closed humeral reduction and pinning under general anesthesia. The patient had a history of allergies after using an oral topical anesthetic spray during a gastroscopy at another hospital. Because of the history that occurred at another hospital long ago, detailed medical records could not be confirmed, and the topical anesthetic was presumed to be lidocaine. The patient stated that urticaria and an itching sensation appeared on the upper body. She had no other medical history, and there were no abnormal findings in the pre-anesthetic patient evaluation, including laboratory and imaging tests and electrocardiography (ECG).

After admission to the operating room, the heart rate (HR) and rhythm were monitored using a 3-lead ECG. Standard monitoring, including noninvasive blood pressure (BP) measurement using an arm cuff and peripheral oxygen saturation (SpO_2_), was also started. The initial BP was 113/64 mmHg, and the HR was 57 beats/min (bpm). Preoxygenation was performed by supplying 6 L of 100% oxygen and 0.2 mg of glycopyrrolate as an anticholinergic and 0.075 mg of palonosetron as an antiemetic were administered intravenously (IV). Remimazolam was prepared as an anesthetic agent at 1 mg/mL and administered at a rate of 12 mg/kg/h for 1 min to induce anesthesia; 10 mg (0.2 mg/kg) were administered. After confirming loss of consciousness, rocuronium 40 mg (0.8 mg/kg) was administered as a neuromuscular blocking agent. Following the administration of rocuronium, the patient developed a skin rash on the chest wall. However, the rash subsided after the administration of 10 mg of dexamethasone. After administration of remifentanil 0.5 µg/kg, tracheal intubation was performed. Immediately after tracheal intubation, noninvasive BP was 32/18 mmHg, HR was 102 bpm, and the skin rash on the entire body worsened, accompanied by swelling. Other than hypotension and tachycardia, no other ECG changes were observed, and transesophageal echocardiography could not be performed due to problems with equipment availability at the institute. No antibiotics had been given; an emergency alarm was activated in the operating room due to suspected rocuronium-induced anaphylaxis, and medical staff were called for assistance. Invasive BP was monitored through radial artery cannulation, and epinephrine was administered while the fluid was loaded through a peripheral 16-gauge IV cannulation. Blood samples were collected through the arterial line immediately after radial artery cannulation; subsequently, arterial blood gas analysis (ABGA) and an acute phase tryptase test were performed. There were no unusual findings in the ABGA results (pH 7.359, PaCO_2_ 44.5 mmHg, PaO_2_ 204.2 mmHg, HCO_3_^−^ 23.7 mmol/L, base excess −2.1 mmol/L, lactate 1.73 mmol/L). After total administration of epinephrine 500 µg IV and crystalloid 1000 mL, the skin rash and BP normalized, and central venous cannulation was performed at the right internal jugular vein. Subsequently, hypotension was observed, and epinephrine was administered at 0.2 µg/kg/min IV. While managing anaphylaxis, the peak airway pressure on the ventilator did not increase, chest auscultation was normal, and SpO_2_ was maintained at 100%. After confirming no signs of airway obstruction, sugammadex 200 mg IV was administered as a neuromuscular blockade reversal agent. Spontaneous breathing was restored, and tracheal extubation was performed. The surgery was canceled, and the patient was discharged to the intensive care unit (ICU). A chest radiography was performed in the ICU and the findings were unremarkable (Supplementary Figure S1).

Within the target of 20% of the initial systolic BP, the epinephrine dose was reduced to 0.1 μg/kg/min after 9 h, and finally, it was discontinued after 40 h. Additional oxygen supply was stopped 17 h after the onset of anaphylaxis. The patient did not complain of respiratory symptoms such as dyspnea when breathing room air. Twenty-six hours after the onset of anaphylaxis, the patient was transferred to the general ward and discharged three days later. The acute phase tryptase level, measured after radial artery cannulation, was 3.9 μg/L.

Four weeks after the anaphylaxis, the patient was readmitted to the allergy department for allergy testing. Intradermal tests were performed with glycopyrrolate, palonosetron, remimazolam, rocuronium, and remifentanil, all administered during anesthesia induction. Additionally, midazolam, which is structurally similar to remimazolam, was evaluated. Intradermal test results were negative for all drugs. A provocation test was performed to exclude the drug as the cause of anaphylaxis. When midazolam was diluted 1:100 in normal saline and administered intravenously at 1 mL, no anaphylactoid symptoms were induced. When remimazolam was diluted to 1 mg/mL in normal saline and administered intravenously at 1 mL, facial edema, rash, and cough occurred 4 min later, and an allergic reaction was observed as SpO_2_ decreased from 99% to 90%. Symptoms improved after IV epinephrine administration. Based on this provocation test, remimazolam was identified as the drug that caused anaphylaxis.

## 3. Discussion

Diagnosing perioperative anaphylaxis can be challenging due to the variety of drugs used, including anesthetics, antibiotics, neuromuscular blockers, and latex. Recently, cases of remimazolam-induced anaphylaxis have been consistently reported. To the best of our knowledge, 11 cases have been reported in six research articles (Table 1) [10,11,12,13,14,15]. Compared with previous cases, the characteristic feature of this case was that the drug for anaphylaxis was finally identified as remimazolam in the provocation test. Generally, in cases of anaphylaxis that occur during anesthesia induction, neuromuscular blockers or antibiotics are most commonly suspected. In previous studies that reported perioperative anaphylaxis, approximately 25–60% and 20–50% of cases were caused by neuromuscular blockers and antibiotics, respectively [16,17]. The present case occurred before antibiotic administration, and we suspected rocuronium-induced anaphylaxis before the allergy test. Through a meticulous approach during the allergy test, we identified remimazolam as the causative drug for anaphylaxis following a provocation test. Therefore, even if the intradermal test was negative for remimazolam, a provocation test was recommended for final confirmation. In further studies, protocols for intradermal tests and provocation tests with remimazolam should be established.

We have summarized the characteristics of remimazolam anaphylaxis by reviewing 12 case reports, including the present case (Table 1). First, most cases of anaphylaxis occurred at the time of induction of general anesthesia, before and after tracheal intubation, when a relatively excessive dose of remimazolam was administered. Therefore, clinicians should monitor patients more carefully when inducing general anesthesia using remimazolam, and the drug should be administered slowly over 1–2 min according to the pharmaceutical label indications. Symptoms of anaphylaxis were reviewed separately for the skin, cardiovascular, and respiratory systems [18]. Skin symptoms have been reported in approximately half of these cases. Cardiovascular symptoms have been reported in most cases with a decrease in BP. Low BP usually occurs at a systolic BP of 30–40 mmHg, and cardiac arrest occurs in severe cases [13,14]. Respiratory symptoms such as hypoxia and airway edema were reported in half of the cases. Respiratory symptoms may tend to be underestimated because most patients are already tracheally intubated. In the present case, no respiratory symptoms occurred at the time of anaphylaxis due to tracheal intubation. However, coughing and hypoxia occurred during the provocation test. For the treatment of anaphylaxis, epinephrine was administered in all cases, and a bolus dose of approximately 250–750 µg IV was administered, except for the extreme values. During anaphylactic management, severe hypertension caused by excessive epinephrine administration may be fatal [19]. The epinephrine dose must be determined through close observation of the patient’s vital signs. The acute phase tryptase levels were significantly higher than the baseline values in the majority of cases, excluding error cases in laboratory tests. Serum tryptase levels are reported to be significantly increased when acute tryptase > ((1.2 × baseline tryptase) + 2) µg/L [20]. In the present case, baseline tryptase levels could not be evaluated because of problems with blood sample handling. However, compared with the acute phase tryptase in other cases, the level in the present case was substantially low. Additionally, even when considering the suggested normal range of tryptase in previous studies as 2.1–9.0 µg/L [12], the acute phase tryptase level of the present case may not have been elevated. Failure to measure the baseline tryptase level is a limitation of the present case. Both acute phase and baseline tryptase levels should be measured, and anaphylaxis cannot be completely ruled out even if the acute phase tryptase level is not significantly elevated, as observed in the present case.

Tsurumi et al. [10] reported that anaphylaxis is induced by a cross-reaction between midazolam and remimazolam. In the present case, allergy testing was performed using midazolam to evaluate the possibility of cross-reactivity. Both intradermal and provocation tests for midazolam were negative. Because the allergy test was negative for midazolam, which has a structure similar to that of remimazolam, the possibility of anaphylaxis induced by the additive dextran 40 rather than remimazolam itself can be considered. Dextran 40 can induce non-IgE-mediated anaphylaxis and may result in negative skin tests [21]. Previous case reports have suggested that in patients with negative skin tests, it is worth considering whether the causative agent of anaphylaxis was dextran 40 rather than remimazolam. Sander et al. [22], in a review article, also reported that anaphylaxis caused by remimazolam might be related to a non-IgE-mediated effect of the excipient dextran-40. Therefore, when conducting allergy tests, the inclusion of remimazolam and dextran 40 is recommended.

This case report had several limitations. First, baseline serum tryptase levels were not evaluated because of blood sample handling issues. In most cases, an increase in tryptase is determined by comparing the acute phase with the baseline value, rather than a specific normal range. Therefore, it is necessary to test the baseline serum tryptase level. However, in the present case, the acute phase tryptase level was much lower than that in other cases, and a significant increase was not confirmed even when the baseline value was measured. Second, because of the lack of agents, allergy tests cannot be performed only for dextran 40. If remimazolam-induced anaphylaxis is suspected, an allergy test for the additive dextran 40 should be performed. Third, a protocol for the provocation test with remimazolam has not been established. In this case, severe allergic symptoms developed during the provocation test. Demoly et al. [23] reported that a previous life-threatening reaction might be considered a contraindication for a drug provocation test. Therefore, based on the present case, further provocation tests for remimazolam should be initiated at lower doses under careful patient monitoring.

## 4. Conclusions

Remimazolam should not be ruled out as the causative drug if anaphylaxis occurs during anesthesia induction. During the allergy test, dextran 40 should also be evaluated and provocation testing for remimazolam should be initiated at a low dose under careful monitoring of the patient’s vital signs. We believe that this case report and literature review will support the establishment of a mechanism for remimazolam anaphylaxis and allergy testing protocols in the future.

## Figures and Tables

**Table 1:** Summary of case reports of remimazolam-induced anaphylaxis.

Case	Age/Sex/ HT (cm)/WT (kg)	Medical/ Allergic History	Operation Plan/Situation	Remimazolam Dose	Skin Symptoms	Cardio-Vascular Symptoms	Respiratory Symptoms	Treatment (Fluid (mL)/Epinephrine/etc.)	Post Anaphylaxis Management	Acute/Base Tryptase (µg/L)	Allergy Test Time/Results	Other
Tsurumi et al., 2021 [5]												
	32/M/162/60	None/None	Wrist fixative removal/GA induction 2 min after RMMZ infu.	6 mg/kg/h for 2 min (total 12 mg)	Facial flush	sBP 49	SpO_2_ 68, cyanosis, laryngeal edema at laryngoscopy	1600/Epi 750 µgIV, 500 µgIM/ chlorpheniramine, hydrocortisone	Intu. ICU TF, Dexmedetomidine, Epi 0.05 µg/kg/m Hydrocortisone, Extu. POD 1 ICU stay 1 d.	5.8/none	4 wks later/ intradermal(+), midazolam intradermal(+)	2nd anaphylaxis attack (20 min later)
Uchida et al., 2022 [6]												
1st	74/M/157/78	HTN, DM/None	Skin graft for a burn/GA induction	4 mg	Can not confirm	sBP 30–40	SpO_2_ 73	2000/Epi 250 µg/Norepinephrine	Epi 0.03–0.2 µg/kg/min	8.3/2.9	No test	
2nd	59/M/176/52	DM/None	Colectomy/GA induction	9 mg	Can not confirm	HR 105 sBP 30–40	Can not confirm	No mention/Epi 300 µg		7.8/4.1	Skin prick(−), intradermal(−)	
Yamaoka et al., 2022 [7]												
	78/M/148/55	None/None	Bowel resection/GA induction	12 mg/kg/h for 1 min	None	HR 120 sBP 40 TEE: LV collapse, hyperdynamic	SpO_2_ 90, Cyanosis, High airway pressure, No breath sounds on auscultation	2500 mL/h /Epi 300 µgIM, Epi 0.3 µg/kg/min	OR Extu. ICU TF, Epi 0.05 µg/kg/min ICU stay 1 d. HLOS POD 8	23.8/4.3	10 wks later/ skin prick(−) intradermal(+) 0.1 mg/mL at 0.02 mL	
Kim el at., 2022 [8]												
1st	65/M/177/75	None/None	Inguinal herniorrhaphy/GA induction, 2–3 min after intu.	12 mg/kg/h for 6.5 min, total 97.5 mg	None	Collapse, ST elevation	None	No mention/Epi	No mentioned	10.1/4.4	Skin prick(−) intradermal(−)	
2nd	69/M/167/64	LC/None	Umbilical herniorrhaphy/GA induction, 2–3 min after intu.	12 mg/kg/h for 6.8 min, total 76.8 mg	None	Collapse, ST elevation	None	No mention/Epi	No mentioned	14.0/6.3	No test	
3rd	66/M/165/53	HCC, COPD, CKD/None	GS, ileostomy take down/GA induction, 2–3 min after intubation	12 mg/kg/h for 5 min, total 56.3 mg	None	Cardiac arrest	None	No mention/Epi 600 µg, CPR (epi 1 mg, atropine 0.5 mg)	No mentioned	12.8/4.2	Skin prick(−) intradermal(−)	
4th	23/F/162/66	Crohn’s/None	Ileocecal resection/GA induction	12 mg/kg/h for 2 min, total 25.6 mg	Facial flush, skin rash	Collapse, tachycardia	Cough, chest tightness	No mention/Epi	No mentioned	2.6/1.5	Skin prick(−) intradermal(−)	Blood sample handling problem
5th	33/F/168/60	None/None	Thyroidectomy/GA induction	12 mg/kg/h for 4.6 min, total 8.3 mg	Facial flush, skin rash	Collapse, tachycardia	Dyspnea	No mention/Epi	No mentioned	9.2/4.2	Skin prick(−) intradermal(−)	
Hasushita et al., 2022 [9]												
	72/M/166/61	HTN/ Acemetacin, kikyosekko(herbal)	Lung op./GA induction, 6 min after tracheal intubation	12 mg	Abdomen erythema	sBP below 50, pulse undetectable, EtCO_2_ 19 mmHg Cardiac arrest	None	No mention/Epi 1 mg/ACLS, Chlorpheniramine, hydrocortisone	Intu., ICU T/F Post 4 h Extu. Vasopressor 15 h ICU stay 1 d HLOS 3 d	8.7/4.8	4 wks later/ intradermal(+) 1:100, 1:10 Dextran 40 skin(−)	
Hu et al., 2023 [10]												
	41/M/165/63	None/None	Colonoscopy/1 min after sedation induction	10 mg	Upper body erythema, swelling	NIBP 77/47, HR 95	Laryngeal stridor, SpO_2_ 91, epiglottic edema, oral secretions, PaCO_2_ 104 mmHg	2000/Epi 50 µg/ Jaw thrust, manual ventilation, LMA	3 h later GW TF	None/none	4 wks later/intradermal(−), midazolam intradermal(+)	Mucosa biopsy, eosinophil infiltrate
Lee et al., 2023 (Present case)												
	51/F/170/52	None/lidocaine	Humeral pinning/GA induction, post tracheal intubation	12 mg/kg/h for 1 min, total 10 mg	Whole body skin rash	sBP 32, HR 102	None	1000/Epi 500 µgIV, 0.2 µg/kg/min/Dexamethasone	OR Extu., ICU TF, Epi 0.2–0.03 µg/kg/min for 40 h ICU stay 1 d HLOS 3 d	3.9/none	4 wks later/intradermal(−), provocation test(+)	Provocation test

BP, SpO_2_, and HR are presented as mmHg, %, and beats per min, respectively. HT, height; WT, weight; sBP, systolic blood pressure; SpO_2_, peripheral oxygen saturation; Epi, epinephrine; IV, intravenous; IM, intramuscular; Intu., tracheal intubation; ICU, intensive care unit; TF, transfer; Extu., tracheal extubation; POD, postoperative days; d., days; wks, weeks; HTN, hypertension; DM, diabetes mellitus; GA, general anesthesia; HR, heart rate; TEE, transesophageal echocardiogram; LV, left ventricle; OR, operating room; HLOS, hospital length of stay; LC, liver cirrhosis; HCC, hepatic cellular carcinoma; COPD, chronic obstructive pulmonary disease; CKD, chronic kidney disease; CPR, cardio-pulmonary resuscitation; EtCO_2_, end-tidal carbon dioxide; ACLS, Advanced Cardiovascular Life Support; NIBP, non-invasive blood pressure; PaCO_2_, partial pressure of carbon dioxide in arterial blood; LMA, laryngeal mask airway; GW, general ward.

## Data Availability

The data used and analyzed during the current study are available from the corresponding author upon reasonable request. The data are not publicly available because of privacy or ethical restrictions.

## Supplementary Materials

The following supporting information can be downloaded at: https://www.mdpi.com/article/10.3390/medicina59111915/s1, Figure S1: The patient’s chest radiography in the intensive care unit after anaphylaxis.
