# Supplementary figures and images for: Remimazolam Anaphylaxis during Induction of General Anesthesia Confirmed by Provocation Test—A Case Report and Literature Review
Source: Medicina (Kaunas). 2023 Oct 30;59(11):1915. doi: 10.3390/medicina59111915 (PMC10673581; doi:10.3390/medicina59111915)

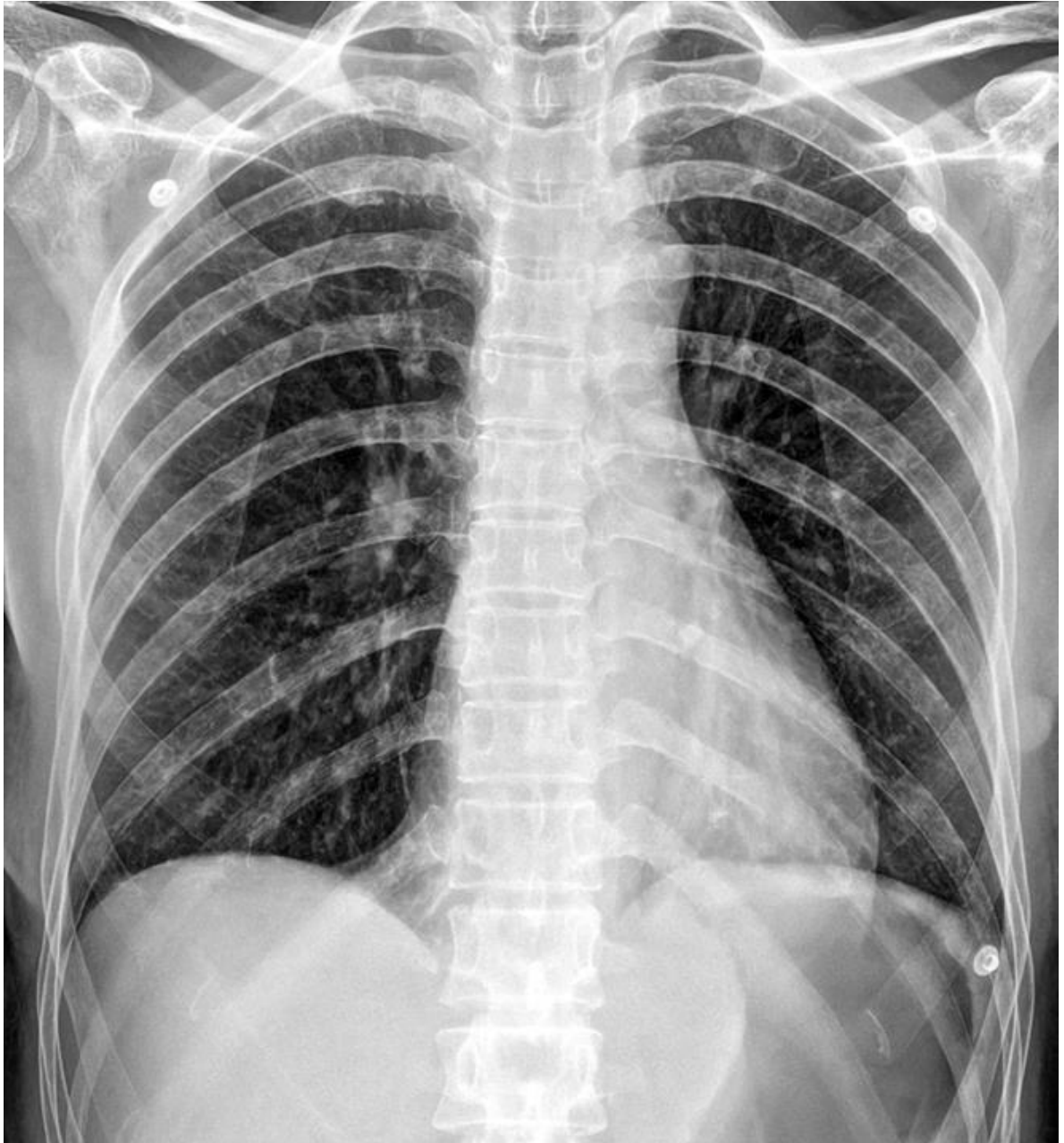

**Figure S1.** The patient's chest radiography in the intensive care unit after anaphylaxis.

Supplement: Supplementary file 1 [file medicina-59-01915-s001.zip › medicina-2656612-supplementary.pdf]
